# VEGF-C induced by TGF- β1 signaling in gastric cancer enhances tumor-induced lymphangiogenesis

**DOI:** 10.1186/s12885-019-5972-y

**Published:** 2019-08-13

**Authors:** Kyung Ho Pak, Ki Cheong Park, Jae-Ho Cheong

**Affiliations:** 1 Department of Surgery, Hallym University Medical Center, Hwasung, Korea; 2 Department of Medicine, Yonsei University Graduate School, Seoul, Korea; 3 Depatment of Surgery, Yonsei University College of Medicine, 50-1 Yonsei-ro, Seodaemun-gu, Seoul, 120-752 Korea; 4 Department of Biochemistry & Molecular Biology, Yonsei University College of Medicine, Seoul, Korea; 5 Brain Korea 21 PLUS Project for Medical Science, Yonsei University College of Medicine, Seoul, Korea

**Keywords:** TGF-β1, VEGF-C, Lymphangiogenesis, Gastric cancer

## Abstract

**Background:**

The role of TGF-β1 in lymph node metastasis and lymphangiogenesis, one of the most important steps of gastric cancer dissemination, is largely unknown. The goal of this study was to investigate the role of TGF-β1 signaling and its molecular mechanisms involved in lymphangiogenesis of gastric cancer.

**Methods:**

Two gastric cell line models, MKN45 and KATOIII, were selected for this study. The protein expression of TGF-β1 pathway molecules and VEGF-C were examined with western blot, or ELISA according to TGF-β1 treatment. To explore whether Smad3 binds to the specific DNA sequences in the *VEGFC* promoter, we performed an electrophoretic mobility shift assay. Lymphatic tube forming assay and gastric cancer xenograft mouse models were also used to elucidate the effect of TGF-β1 on lymphangiogenesis.

**Results:**

TGF-β1 induced the activation of Smad2/3 and Smad pathway-modulated VEGF-C expression in gastric cancer cell line models. Phosphorylated and activated Smad3 in the nucleus bound to the promoter of *VEGFC* in KATO III cells*.* Of note, in MKN45 cells, the Smad-independent AKT pathway was also activated in response to TGF-β1 and induced VEGF-C expression. Inhibition of TGF-β1 signaling down-regulated the expression of VEGF-C. We also confirmed, through tube forming assay and tumor xenograft mouse model, that TGF-β1 increased lymphatic formation, while TGF-β1 inhibition blocked lymphangiogenesis.

**Conclusion:**

Smad-dependent and -independent TGF-β1 pathways induce VEGF-C, which make lymphangiogenesis around tumor. These findings suggest that TGF-β might be a potential therapeutic target for preventing gastric cancer progression and dissemination.

**Electronic supplementary material:**

The online version of this article (10.1186/s12885-019-5972-y) contains supplementary material, which is available to authorized users.

## Background

Tumor-induced lymphangiogenesis has been studied to have an association with lymph node metastasis and poor prognosis of cancer patients [1–7]. It is regard as not a simple route of cancer to regional lymph node, but behaves an active role for metastasis. The most well studied mechanism of lymphangiogenesis is VEGF-C/D and VEGFR3 interacting axis between cancer cells and lymphatic endothelial cells [8, 9].

For gastric cancer, lymph node metastasis and lymphangiogenesis is one most significant prognostic factor determining the clinical outcomes after curative intent treatment. Recent multicenter transcriptome studies [10] and The Cancer Genome Atlas study [11] showed that TGF-β may play an important role in gastric cancer biology and progression. Until now, TGF-β can lead bad prognosis of cancer patients by promoting epithelial-mesenchymal transition (EMT), leading to invasion and metastasis [12, 13]. However, the effect of TGF-β for lymphangigoenesis, which is the one of the most important steps during metastasis of cancer, has been largely unknown.

Here, we investigated the role and molecular signaling mechanism of TGF-β1 for lymphangiogenesis in gastric cancer. We elucidated that TGF-β1 promotes VEGF-C production through Smad-dependent way or Smad-independent one, according to each cell type, which leads to lymphangiogenesis in gastric cancer in vitro and in vivo experimental model. Our study provide insight for understanding TGF- β cancer biology and a new target for cancer treatment.

## Methods

### Cell culture

The human gastric cancer cell lines AGS, MKN28, MKN45, NCI-N87, SK4, KATOIII, Hs746T, and YCCs were maintained in RPMI 1640 medium (Hyclone, South Logan, Utah) containing 10% fetal bovine serum, 100 U/ml of penicillin sodium, and 100 μg/ml of streptomycin sulfate at 37 °C in a humidified incubator containing 5% CO2. Cultured human lymphatic endothelial cells (HLECs) were purchased from Promo Cell (Promo Cell, Heidelberg, Germany). This cell line was maintained in complete medium (Endothelial cell growth medium 1; Promo Cell, Heidelberg, Germany) on gelatin-coated dishes. HLECs were used between passages 5 and 8.

Human gastric cell lines MKN45 and KATOIII were obtained in May 2007 from American Type Culture Collection (ATCC, Manassas, VA, USA). AGS, MKN28, NCI-N87 and Hs746T were purchased in March 2007 from the Korean Cell Line Bank (Seoul, Korea). SK4 cells were a kind gift in May in 2009 from Dr. Julie Izzo (MD. Anderson Cancer Center, Texas, USA). YCC-series cell lines were obtained from in September 2006 the Song-Dang Institute for Cancer Research, Yonsei University College of Medicine (Seoul, Korea). The identifies of all cell lines were validated by short tandem repeat (STR) DNA fingerprinting using the AmpFISTR identifier kit, according to the manufacturer’s guidelines (Applied Biosystems; 4322288) at the Characterized Cell Line Core Facility. The STR profiles were compared with known ATCC fingerprints (ATCC.org) and to the Cell Line Integrated Molecular Authentication (CLIMA) database version 0.1.200808 (http://bioinformatics.hsanmartino.it/clima2/; Nucleic Acids Research 37:D925-D932 PMCID: PMC2686526). *Mycoplasma* contamination was checked by *Mycoplasma* PCR Detection Kit (Sigma-Aldrich; MP0035) to ensure that there was no mycoplasma contamination before and after the study.

### Western blot analysis

Cells were washed cold PBS and lysed on ice with protein extraction buffer (Pro-Prep, iNtRON Biotechnology, Seoul, Korea). Equal amounts of protein were loaded onto a sodium dodecyl sulfate-polyacrylamide gel (12% polyacrylamide) and transferred to polyvinylidene floride (PVDF) membrane. The membranes were blocked with 5% nonfat milk in TBS-T and incubated with appropriate concentrations of primary antibodies anti-TGFβ receptor II (TβR2), Smad3, P-Erk, P-Akt, Akt, P-Rho, Rho, VEGF-C (dilution 1:1000; cell signaling Technology, Massachusetts, USA) and anti-β-actin (dilution 1:1000; Sigma-Aldrich, USA) antibodies were diluted in TBS-T (TBS/Tween 20: 2% skim milk). The appropriate secondary antibodies were applied (dilution 1:5000, horseradish peroxidase-conjugated anti-rabbit and anti-mouse) at room temperature for 1 h. Labeled bands were detected by enhanced chemiluminescence (ECL; ThermoScientific, USA).

### Electrophoretic mobility shift assay (EMSA)

The DNA binding activity of Smad3 against the *VEGFC* promoter was studied with a ^32^P-labeled oligonucleotide encoding the Smad3 transcription factor binding sites found in the *VEGFC* promoter region [14–16]. We first used the Alibaba 2.1 algorithms to search the TRANSFAC database to identify potential Smad3-binding sites to VEGFC promoter. This search revealed the presence of Smad3 binding sites to *VEGFC* promoter. Double-stranded oligonucleotides including the consensus-binding site for Smad3 sense: GTCGGCCAGC -CACTCGCATTGTGACTA, anti-sense: TAGTCACAATGCGAGTGGCTGGCCGAC and Mutant Smad3 sense: GTCGCGGAGCCACTCGCTAACTGACTG, anti-sense: CAGTCA -GTTAGCGAGTGGCTCCGCGAC were 5′ end-labeled with polynucleotide kinase and ɣ-^32^P-dATP. Details are described in our previous articles [17].

### Tube formation assay

HLECs (1 × 10^5^) were cultured in a 24-well plate coated with 150 μl of Growth factor-diminished Matrigel in MV1 medium for cell attachment for 1 h. The MV1 medium was substituted with conditional medium and continuous cell culture for 24 h. Then, tube length was calculated after 8 h by checking the total cumulative tube length in three random microscopic fields with NIH ImageJ1.44 image analysis software, which is available at https://imagej.nih.gov/ij/. The original magnification used was × 100.

### Enzyme-linked immunosorbent assay (ELISA)

Protein contents in culture medium were determined with Quantikine Immunoassay systems for human VEGF-C enzyme-linked immunosorbent assay (ELISA) kit (R&D Systems, Minneapolis, MN, USA). Content of target protein in culture medium was expressed as the quantity of protein secreted from 10,000 cells for 24 h.

### Human GC (gastric cancer) cell lines xenograft model

The human GC cell lines MKN45 and KATOIII (1.5 × 10^6^ cells/mouse) were cultured in vitro and then injected subcutaneously into the upper left flank region of female BALB/c nude mice. After 10 days, tumor-bearing mice were grouped randomly (*n* = 8/group) and treated daily oral dosing at 75 mg/kg TGF-β inhibitor (LY2157299). Tumor size was measured every other day using calipers. Tumor volume was estimated using the following formula: (LxS^2^)/2 (where L, longest diameter; S, shortest diameter) [18]. Female BALB/c nude mice were purchased from the Orient Animal Co. Ltd. (Seongnam, Gyeonggi-do, Korea). Mice were sacrificed by CO2 asphyxiation, and tumors were excised. All animals were sustained under specific pathogen-free (SPF) conditions. All experiments received the Animal Experiment Committee of Yonsei University and the Institutional Review Board of Severance Hospital, Yonsei University (4–2012-0427) approval.

### Statistical analysis

Values are expressed as means ± S.D. Mann-Whitney test was used to evaluate the data. For cell tube assay analysis, One-Way ANOVA was used to assess statistical significance. Differences were rated to be statistically significant at *P* < 0.05. All analyses were excuted with SPSS 21 software (SPSS, Chicago, IL).

## Results

### GC cells which show TGF-β1, TβR2 and VEGF-C are selected as the model cell lines

**Fig. 1:**
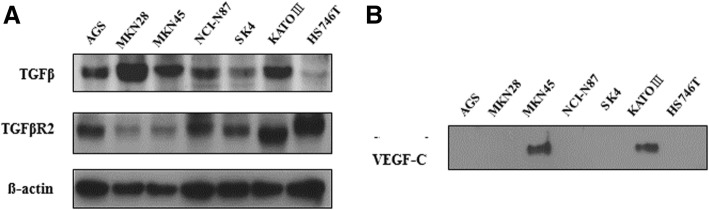
TGF-β1, TGF-β1 receptor II, and VEGF-C expression in gastric cancer cells. Among gastric cancer cell lines, MNK45 and KATOIII were selected as a model for the regulation of TGF-β1 on lymphangiogenesis. These two cell lines expressed TGF-β1, TGF-β receptor II (**a**), and VEGF-C (**b**). TβR2, TGF-β receptor 2

To select model cell lines suitable for testing the hypothesis, we examined the expression of TGF-β1, TβR2 and VEGF-C in a panel of gastric cancer cell lines (AGS, MKN28, MKN45, NCI-N87, SK4, KATOIII, HS746T) by western blot analysis. TGF-β1 and TGF-β1 receptor II were expressed in all seven gastric cancer cell lines examined (Fig. 1a), whereas VEGF-C was expressed only in MKN45 and KATOIII (Fig. 1b) cell lines. Based on these results, we selected MKN45 and KATOIII as model cell lines for improving the role of TGF-β1 signaling on lymphangiogenesis.

### GC cells secrete TGF-β1 and shows paracrine effect

We further investigated additional gastric cancer cell lines and found that YCC2 and YCC3 only express a significant amount of TβR2, but not TGF-β1 (Additional file 1: Figure S1A). We ascertained the existence of TGF-β1 in conditioned media of MKN45 and KATOIII but not in YCC2 (Additional file 1: Figure S1B). We noticed that Smad2 and Smad3, the receptor activated Smads (R-Smads), were phosphorylated and activated in YCC2 cells when treated with the conditioned media of MKN45 and KATOIII (Additional file 1: Figure S1C). Therefore, we confirmed that our gastric cancer cell-line models (MKN45 and KATOIII) produce TGF-β1, which can regulate other cells in paracrine fashion.

### GC cells are confirmed to have a normal response to TGF-β1 and its inhibitor

To confirm the validity of these model cell lines for TGF-β1 responsiveness, MKN45 and KATOIII cells were treated with TGF-β1 (10 ng/ml) and were analyzed for target gene expression. As shown in Additional file 2: Figure S2A, the mRNA expression of *twist1*, a well-established target gene of the TGF-β1 signaling pathway, was significantly induced by TGF-β1 treatment in both cell lines. In addition, the corresponding increase of phosphorylation of Smad3 was evident in TGF-β1-treated cells (Additional file 2: Figure S2B). Further, the treatment of TGF-β1 receptor I inhibitor (LY2157299) abrogated the effect of TGF-β1-induced phosphorylation of Smad3, confirming intact and functioning TGF-β1 signaling in these cell lines (Additional file 2: Figure S2C).

### GC cells transduce TGF-β1 signaling through translocation of cytosolic P-Smad3 into nucleus

**Fig. 2:**
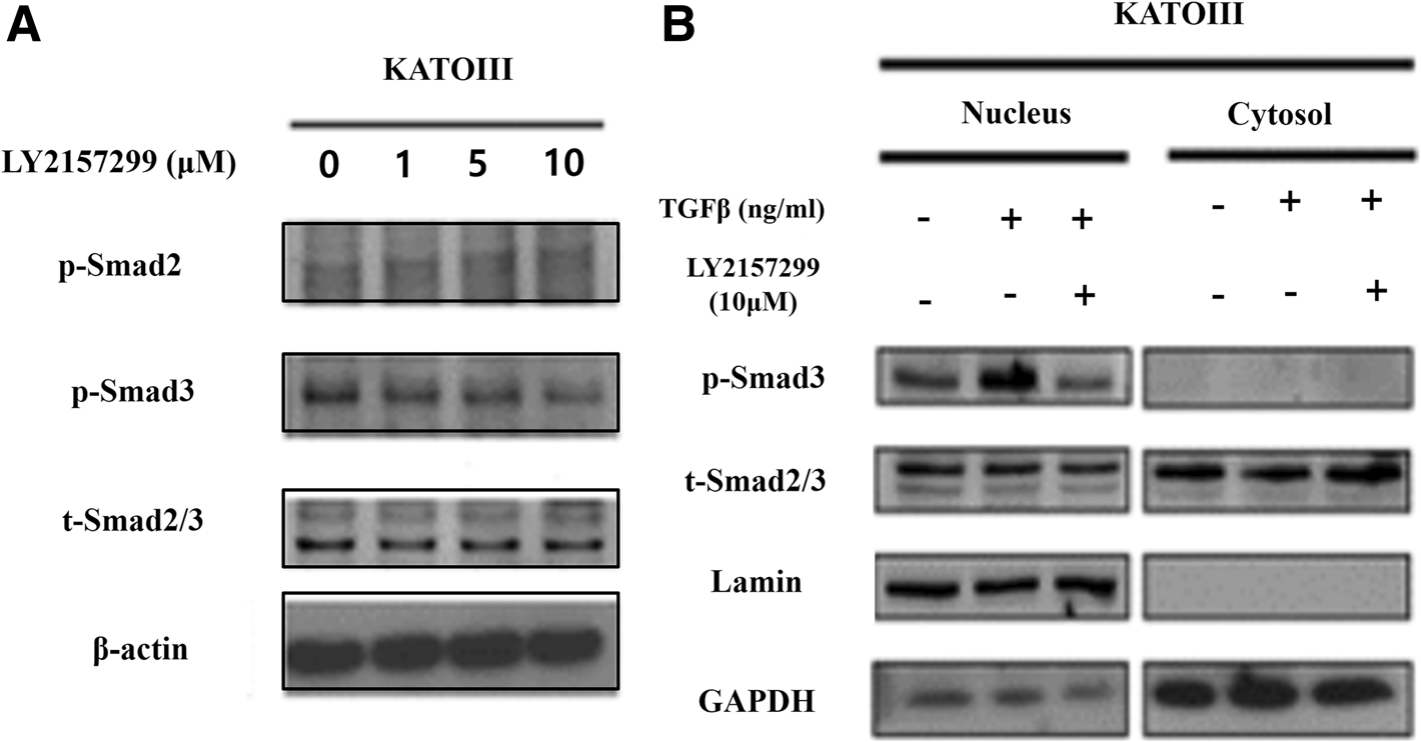
Smad-dependent pathway of TGF-β1 signaling. **a** LY2157299 suppressed the expression of phospho-Smad3 in KATOIII. **b** The change in p-Smad3 expression in response to TGF-β1 in the cytosol and nucleus. In KATOIII cells, the amount of nucleus p-Smad was increased in response to TGF-β1, while decreased in response to LY2157299. Lamin and GAPDH were used as a loading controls in the nucleus and cytosol, respectively

To investigate the effect of TGF-β1 on the expression of VEGF-C, we treated KATOIII cells with TGF-β1 and/or TGF-β1 receptor I inhibitor (LY2157299), as indicated. In KATOIII cells, the expression of phosphorylated form P-Smad3, not P-Smad2, was down-regulated in response to LY2157299. Furthermore, it was repressed in dose-dependent manner (Fig. 2a). To examine whether activated Smad3 was translocated to nuclear from cytoplasm in response to TGF-β1, we analyzed the expression level of both P-Smad3 and total form Smad3 in nucleus and cytosol according to the treatment of TGF-β1 and LY2157299. The expression of P-Smad3 in the nucleus was increased with TGF-β1, while it was decreased with LY2157299 (Fig. 2b).

### Translocated P-Smad3 binds directly to the promoter region of *VEGF-C*

**Fig. 3:**
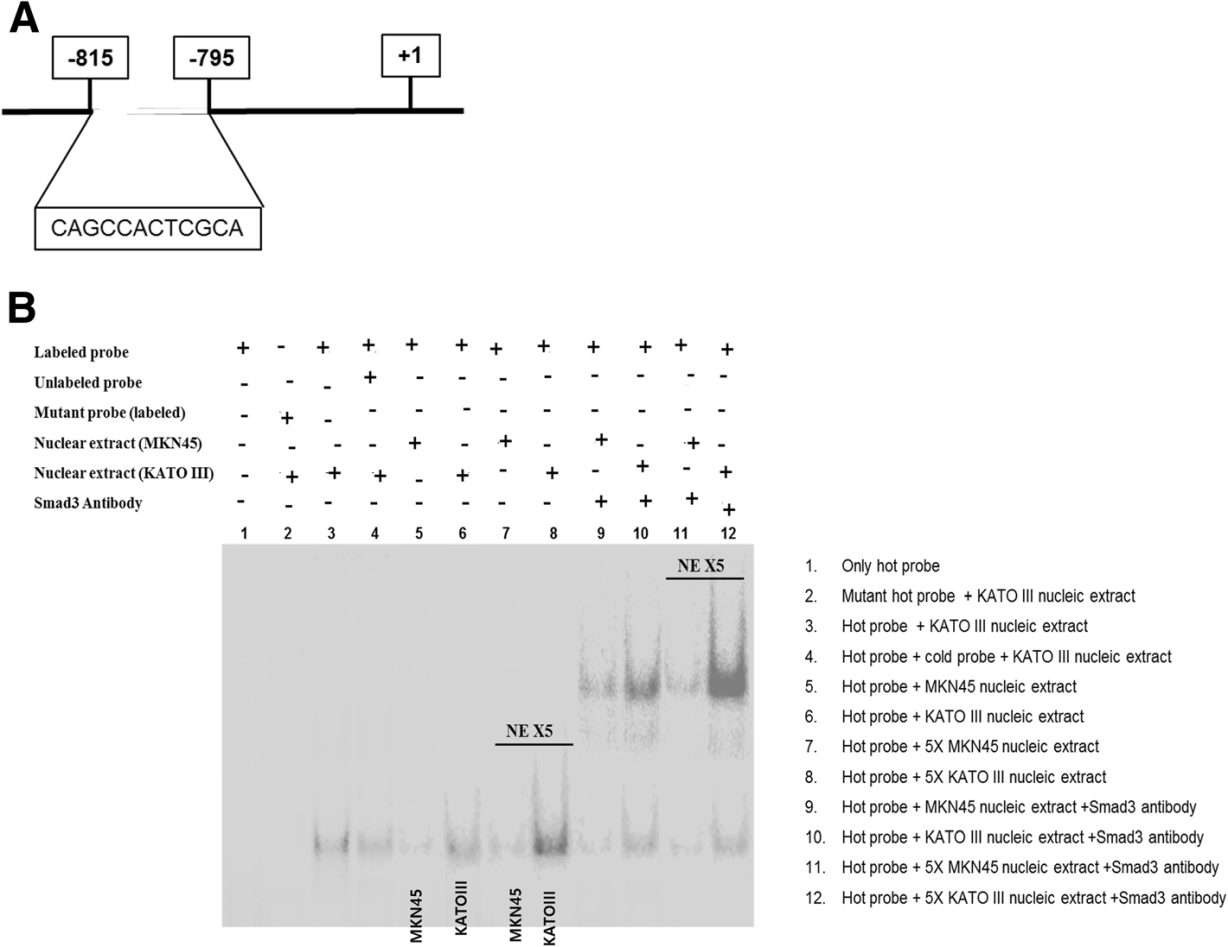
EMSA between Smad3 and *VEGFC*. **a** The binding site of Smad3 to the promoter of *VEGFC.* **b** KATOIII cells (lane 6 and 8) showed significantly increased binding activity between Smad3 and the *VEGFC* promoter region compared to MKN45 cells (lane 5 and 7). NE, nuclear extract

To elucidate whether TGF-β1 signaling-induced lymphangiogenesis is mediated through increased transcription of VEGF-C, we first investigated the interaction between transcription regulator Smad3 and the promoter region of *VEGFC*. A few Smad3 binding sites have been identified in the *VEGFC* promoter region. We carried out an EMSA with a ^32^P-labeled oligonucleotide containing Smad3 binding sites found in the *VEGFC* promoter (Fig. 3a). Labeled Smad3 probe-nuclear extract (from the MKN45 and KATOIII cells) complexes produced two bands. The specificity of the EMSA result was confirmed by complete inhibition of Smad3 DNA binding by excess labeled and unlabeled Smad3 (lane 1, 4, Fig. 3b). In addition, a similar amount of mutated Smad3 probe also failed to bind to the Smad3 transcription complex (lane 2, Fig. 3b).

In KATOIII cells (lane 3, 6, Fig. 3b) Smad3 binding activity to the *VEGFC* promoter regions significantly increased compared to that in MKN45 cells (lane 5, Fig. 3b). The binding signals were increased with five times more nuclear extract alone (lane 7 and 8, Fig. 3b). To assure the binding interaction between Smad3 and the promoter of *VEGFC*, a super-shift assay with anti-Smad3 antibody was performed and showed more potentiated signals (lane 9 and 10, Fig. 3b). Furthermore, that response was potentiated by more concentrated nucleic extract (lane 11 and 12, Fig. 3b). Together, these results demonstrate that Smad3 can bind to the promoter of the *VEGFC* gene.

### Inhibition of TGF-β1 signaling suppresses lymphangiogenic VEGF-C expression

**Fig. 4:**
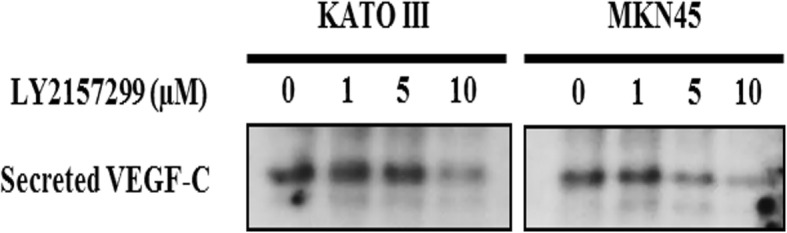
The expression of VEGF-C in response to TGF-β1 receptor I inhibitor. TβR1 inhibitor, TGF-β receptor 1 inhibitor

Next, we examined whether the inhibition of TGF-β1 signaling in model cell lines suppresses VEGF-C expression. By western blot analysis of conditioned media of KATO III and MKN45 cells, we demonstrated that the protein level of VEGF-C is decreased when treated with LY2157299 (Fig. 4)**.** Based on these results, we confirmed that TGF-β1 signaling promotes VEGF-C expression in gastric cancer cells.

### Some GC cells are capable of transducing TGF-β1 signaling through Smad-independent pathway, especially AKT pathway

**Fig. 5:**
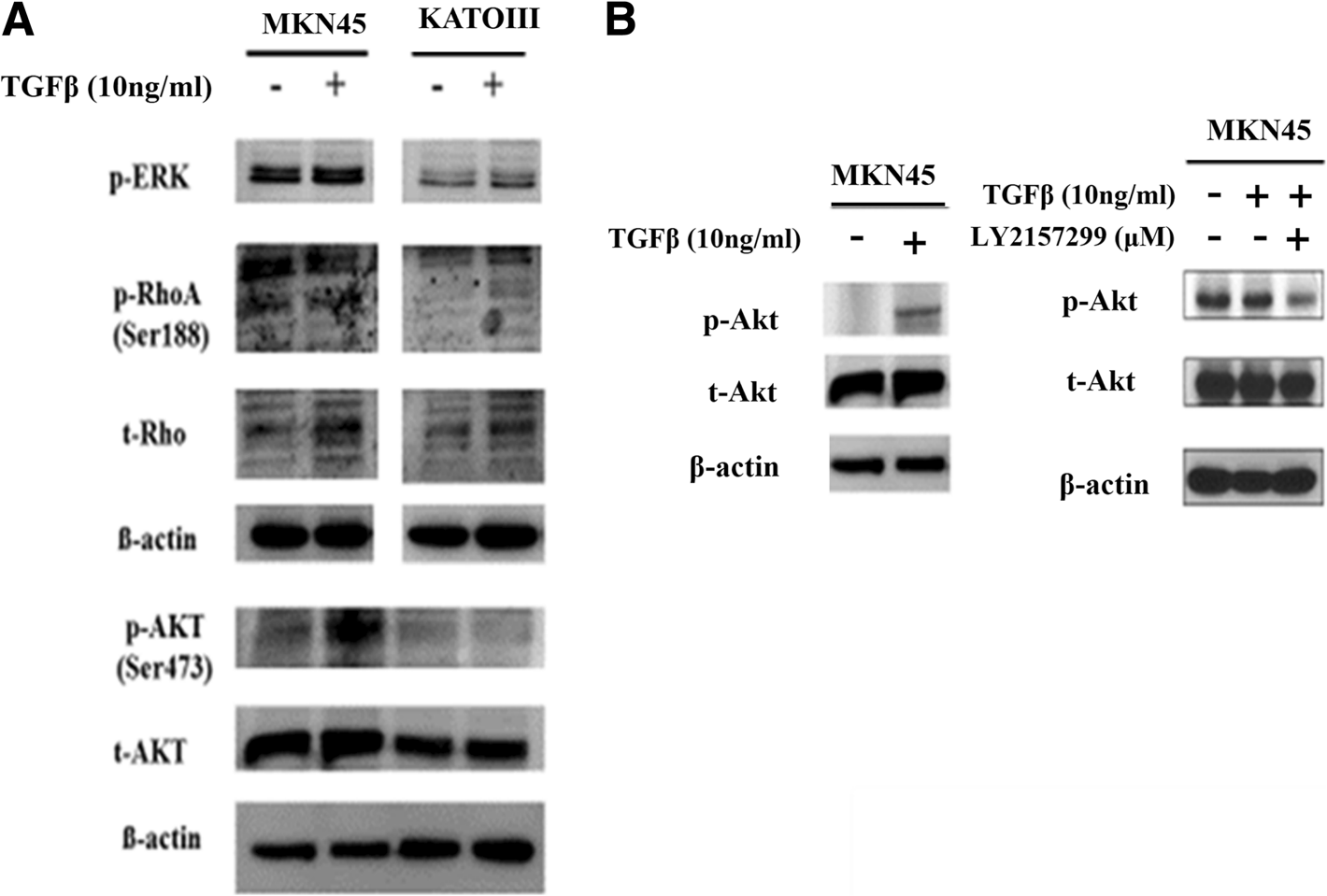
Smad-independent pathway of TGF-β1 signaling. **a** Only p-Akt was remarkably increased in response to TGF-β1 in MKN45, not in KATOIII cells. **b** Phospho-Akt was increased in response to TGF-β1 but decreased in response to LY2157299

According to the EMSA results, the binding interaction between Smad3 and the *VEGFC* promoter region is weaker in MKN45 than KATOIII cells. These findings may suggest the existence of other TGF-β1 signaling pathways for VEGF-C activation in MKN45, which are different from the Smad-dependent pathway. Therefore, we examined Smad-independent signaling pathway molecules. Among them, phospho-Akt (P-Akt) was remarkably induced in response to TGF-β1 treatment in only MKN45 cells, not KATOIII cells (Fig. 5a). P-Akt was increased when treated with TGF-β1, but decreased when treated with its inhibitor LY2157299 in MKN45 cells (Fig. 5b). Taken together, TGF-β1 signals may be transduced through Smad-dependent and Smad-independent pathways according to specific gastric cancer cell line.

### TGF-β1 proliferates lymphatic endothelial cells via VEGF-C production

**Fig. 6:**
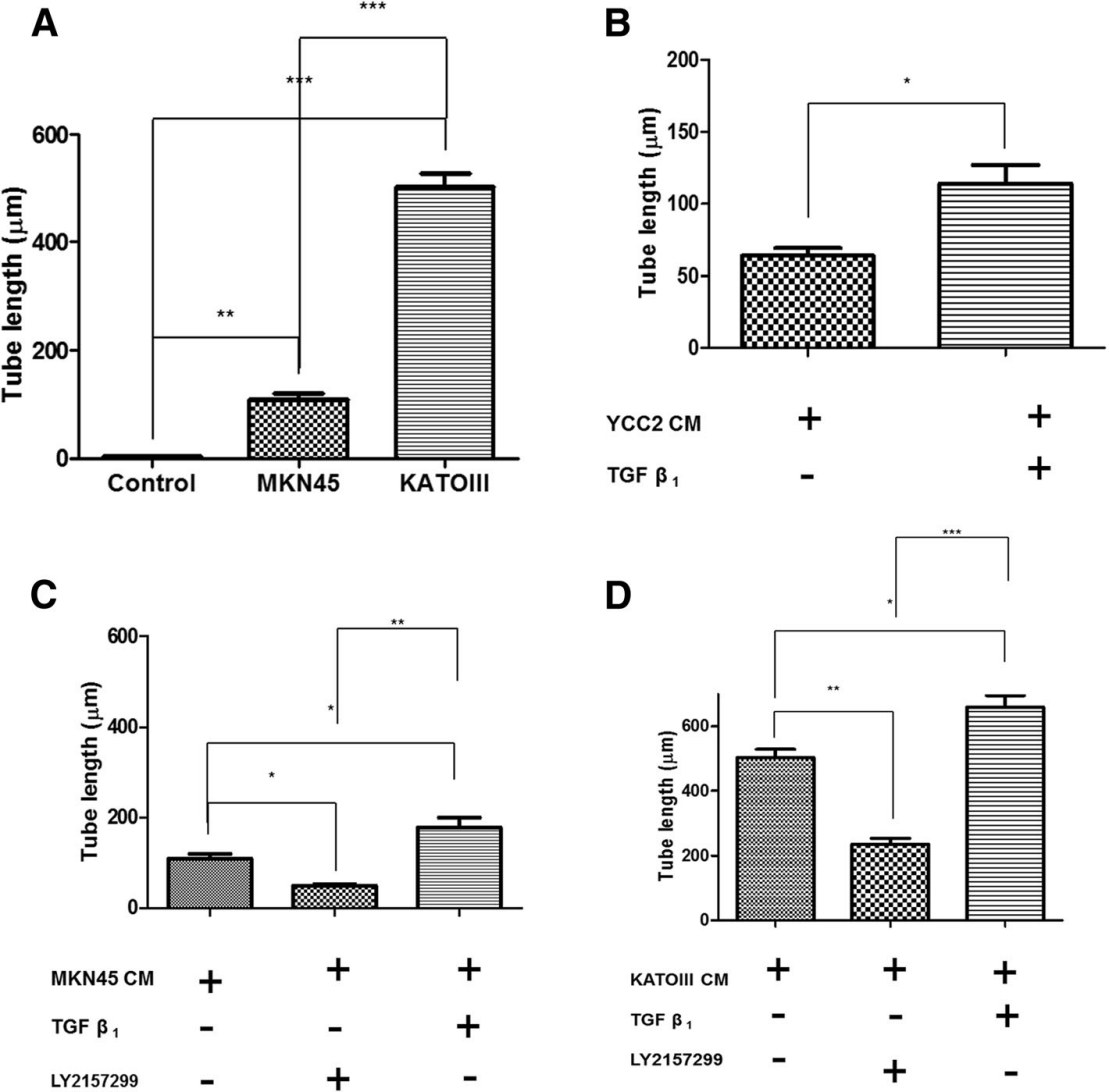
The quantitation of tube formation of HLECs. **a** The length of its tube was longer in MKN45- and KATOIII-conditioned media than in HLEC alone. **b** With YCC2-conditioned media, only TGF receptor-expressing cell line showed increased tube length with TGF-β1. **c-d** With MKN45 and KATOIII-conditioned media, the tube length was increased for TGF-β1 and deceased with TβR1 inhibitor. TβR1, TGF-β receptor 1. (*P < 0.05, **P < 0.01, ***P < 0.001, One-Way ANOVA)

Next, we validated the effect of TGF-β1 signaling on lymphangiogenesis by conducting a lymphatic endothelial cell tube formation assay. HLECs were cultured in the conditioned media of MKN45 and KATOIII cells. After 18 h of culture, we noticed the formation of a tubular structure of HLECs in MKN45- and KATOIII-conditioned media compared to HLECs alone or in YCC2-conditioned media. The length of HLECs in MKN45- and KATOIII-conditioned media was increased compared to HLECs alone or with YCC2-conditioned media (Additional file 3: Figure S3A). The tube length was decreased with in response to TGF-β receptor 1 inhibitor, LY2157299 (Additional file 3: Figure S3B). Quantitation of the results was confirmed by measuring the length of tube formation of HLECs. The length of its tube was longer in MKN45- and KATOIII-conditioned media than in control media (*P* < 0.01, 0.001 each) (Fig. 6a). In YCC2-conditioned media, the length of tube was increased with addition of TGF-β1 (*P* < 0.05) (Fig. 6b). In MKN45 and KATOIII-conditioned media, the tube length was increased with TGF-β1 (*P* < 0.05) and deceased with TβR1 inhibitor, LY2157299 (*P* < 0.05 in MKN45, *P* < 0.01 in KATOIII) (Fig. 6c, d). The expression level of secreted VEGF-C in YCC2-, MKN45-, and KATOIII-conditioned media according to the treatment of TGF-β1 and LY2157299 was also investigated by ELISA analysis. The level of VEGF-C was increased with TGF-β1 in YCC2-conditioned media (*P* < 0.001) (Additional file 4: Figure S4A). The level of VEGF-C was increased with TGF-β1 (*P* < 0.01 in MKN45, *P* < 0.05 in KATOIII), but decreased with LY2157299 (*P* < 0.05 in MKN45, *P* < 0.01 in KATOIII) (Additional file 4: Figure S4B, C**)**.

### TGF-β_1_ receptor inhibitor suppresses gastric cancer progression and lymphangiogenesis in mouse xenograft tumor models

**Fig. 7:**
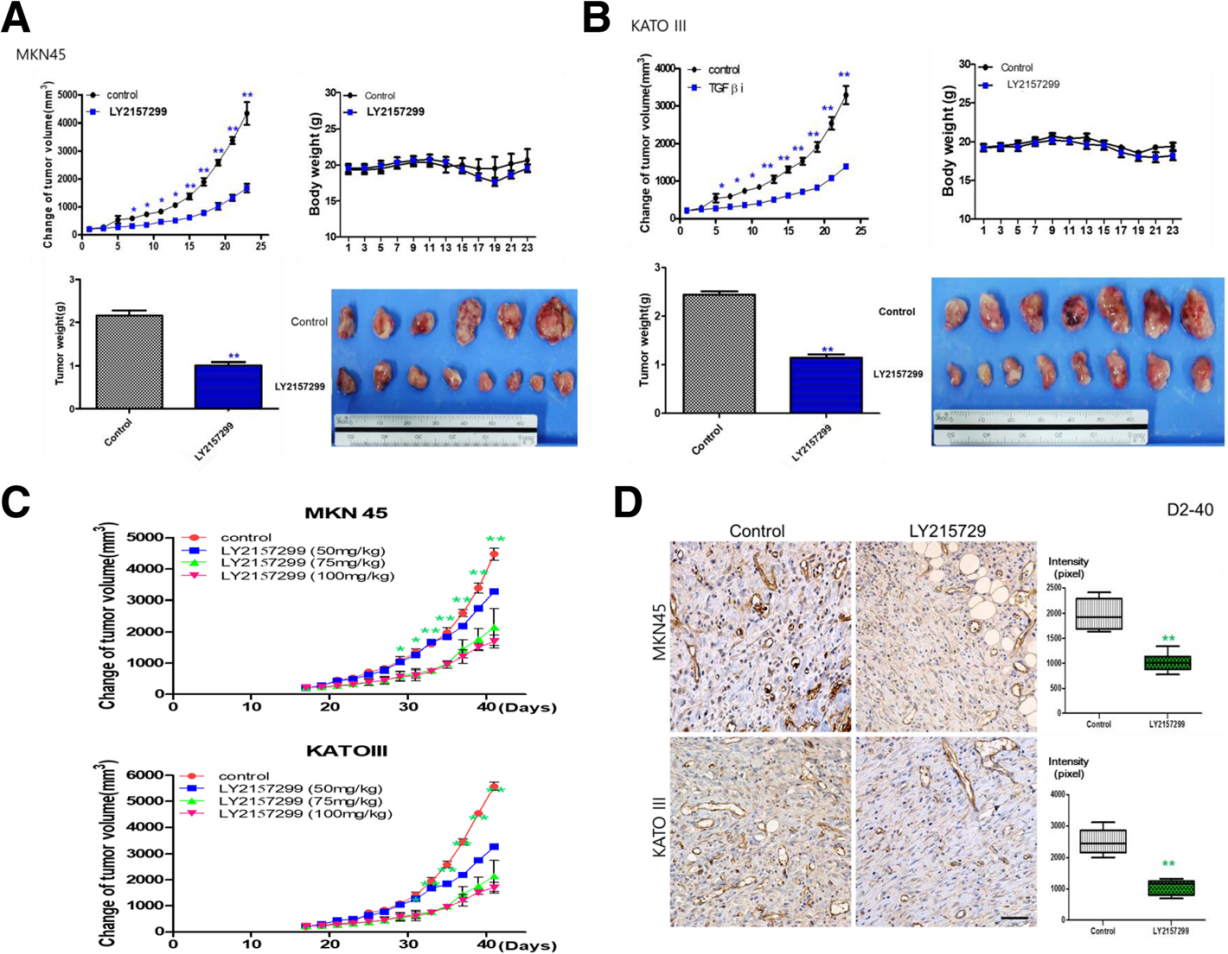
TGF- β1 promotes tumor growth and lymphangiogenesis in mouse xenograft model. **a** TGF-β1 inhibitor reduces tumor volume in MNK45 xenograft mouse model. **b** TGF-β1 inhibitor reduces tumor volume in KATOIII xenograft mouse model. **c** TGF-β1 inhibitor reduces the volume of xenograft model in dose-dependent manner. **d** Lymphovascular density with D2–40 immunohistochemistry. LY2157299 inhibits lymphangigoenesis in both types of gastric cancer tissue. (*P < 0.05, **P < 0.01, Mann-Whitney test)

To study the anti-tumor effect of TGF-β_1_ receptor inhibitor in vivo, we developed mouse xenograft tumor models using MKN45 and KATOIII cell lines. LY2157299 treatment showed significant suppression of xenografted tumors (*P* < 0.01) (Fig. 7a, b). Furthermore, it decreased tumor volume in dose-dependent manner (*P* < 0.01) (Fig. 7c). To confirm the suppressive effect of tumor lymphangiogenesis by TGF-β_1_ receptor inhibitor, we analyzed D2–40 (the marker of small lymphatic vessels) expressions in tumor tissues by immunohistochemical analysis. LY2157299 treated-tumor tissues showed much lower expressions of D2–40 than control (*P* < 0.01) (Fig. 7d).

## Discussion

Our study shows that TGF-β1 signaling which is activated in gastric cancer cells can transcriptionally induce VEGF-C expression, which leads to enhanced tube formation of HLECs in vitro and in vivo. Furthermore, the affecting signaling pathway of TGF-β1 for VEGF-C regulation can be transduced through Smad-dependent or Smad-independent manner in according to different types of gastric cancer cells. The results of this study were supported with the experiment of HLEC tube formation assay and ELISA of secreted VEGF-C in the conditioned media of gastric cancer cells. In addition, gastric cancer cell-line mouse xenograft model showed increased lymphangiogenesis with TGF-β1, while decreased lymphangiogenesis with TGF-β1 inhibitor. This study is the first report to demonstrate the mechanism of how TGF-β1 affects lymphangiogenesis in gastric cancer cell models through investigating both Smad-dependent and Smad-independent pathways.

TGF-β signaling consists of two pathways. One is canonical Smad-dependent pathway and the other is Smad-independent one. TGF-β binds to TGF-β receptor II and I, which phosphorylates Smad2/3. It forms complex with Smad4 in cytosol, which can move into nucleus. Then, it plays a role as transcriptionally regulatory factor for target gene. The Smad-independent pathway includes the phosphoinositol-3 kinase (PI3K), mitogen-activated protein kinase (MAPK), and small guanosine triphosphatase (GTPase) pathways.

Recent studies in gastric cancer [10, 11], colon cancer [19] and hepatocellular carcinoma [20, 21] have brought great interest in the role of TGF-β on gastric cancer biology. TGF-β1 has been known to have a distinct biphasic role in tumor progression. It functions as a tumor suppressor in early stages of cancer, while a tumor promoter in late stages [12, 13]. This dual role of TGF-β1 on tumor biology makes it difficult to understand its mechanism and to apply it therapeutically to a clinical setting. In addition, the reciprocal relation between cancer and surrounding stromal cells, such as lymphocytes, macrophage, fibroblast, etc., makes the situation more complex and complicated [22]. TGF-β1 has many oncogenic effects like epithelial-mesenchymal transition (EMT), angiogenesis, evasion of immune surveillance and stem cell maintenance. However, the role of TGF-β1 on lymphangigoenesis is largely unknown, despite the fact that lymphangiogenesis and lymph node metastasis is one most significant prognostic factor in many types of cancers. Recent some studies suggest that TGF-β1 might upregulate VEGF-C expression in renal tubular cell, implying that TGF-β1 might contribute to tumor lymphangiogenesis [23].

The data in this study suggest that TGF-β1 signaling can upregulate VEGF-C expression, which leads to lymphangiogenesis in gastric cancer. The signals of TGF-β1 to transcriptional induction of VEGF-C can be mediated via canonical Smad3 in some gastric cancer cells, while it can also be conveyed via the non-canonical Smad-independent Akt pathway. However, LY2157299 (Galunisertib, Eli Lilly) which is used in this study as a TβR1 small molecule inhibitor might be thought to inhibit both Smad-dependent and Smad-independent pathways, because both pathways are downstream of the common upstream complex of TGF-β1 and TβR1 [12]. Combination of galunisertib with PD-L1 blockade resulted in improved tumor growth inhibition of hepatocellular carcinoma [21]. Thus, our results support that LY2157299 can be a new therapeutic agent against gastric cancer progression and metastasis.

## Conclusion

Smad-dependent and -independent TGF-β1 pathways induce VEGF-C, which make lymphangiogenesis around tumor. These findings suggest that TGF-β might be a potential therapeutic target for preventing gastric cancer progression and dissemination.

## Additional files

Additional file 1: **Figure S1** Paracrine regulation of TGF-β1. **(A-B)** YCC2 was selected as a paracrine model of TGF-β1 production. **(C)** Smads were detected only in the total cell lysate of YCC2, which was cultured in the conditioned media of MKN45 and KATOIII cells. (TIF 945 kb)

Additional file 2: **Figure S2** Activated TGF-β1 signaling in MKN45 and KATOIII gastric cancer cells The expression of twist I (A) and p-Smad3 (B) were enhanced in responding to TGF-β1, but was decreased at TGF-β1 receptor inhibitor (LY2157299) (C). (TIF 931 kb)

Additional file 3: **Figure S3** Lymphatic endothelial cell (HLEC) growth in the conditioned media of gastric cancer cells. The growth of HLEC in MKN45- and KATOIII-conditioned media was increased compared to HLECs alone or with YCC2-conditioned media. However, tube formation was decreased in responding to TGF receptor I inhibitor. All photos were taken after 8 h of culture. CM, conditioned media; TβR1 inh., TGF-β receptor 1 inhibitor. (TIF 3336 kb)

Additional file 4: **Figure S4** The level of VEGF-C in the conditioned media of gastric cancer cell lines. **(A)** YCC 2-conditioned media resulted in an increased level of VEGF-C with TGF-β1. **(B-D)** The level of VEGF-C was increased with TGF-β1, but decreased with TβR1 inhibitor in MKN45- and KATOIII-conditioned media treatments. TβR1, TGF-β receptor 1. *P < 0.05, **P < 0.01, ***P < 0.001, One-ANOVA test). (TIF 871 kb)

## Data Availability

The datasets used and analyzed during the current study are available from the corresponding author on reasonable request.

## Abbreviations

ELISA: Enzyme-linked immunosorbent assay
EMSA: Electrophoretic mobility shift assay
HLECs: Human lymphatic endothelial cells
PVDF: Polyvinylidene floride
TGF: Transforming growth factor
TβR: Transforming growth factor-beta receptor
VEGF: Vascular endothelial growth factor
VEGFR: Vascular endothelial growth factor receptor

## References

[1] Gombos Z, Xu X, Chu CS, Zhang PJ, Acs G (2005). Peritumoral lymphatic vessel density and vascular endothelial growth factor C expression in early-stage squamous cell carcinoma of the uterine cervix. Clin Cancer Res.

[2] Liang P, Hong JW, Ubukata H, Liu HR, Watanabe Y, Katano M, Motohashi G, Kasuga T, Nakada I, Tabuchi T (2006). Increased density and diameter of lymphatic microvessels correlate with lymph node metastasis in early stage invasive colorectal carcinoma. Virchows Arch.

[3] Roma AA, Magi-Galluzzi C, Kral MA, Jin TT, Klein EA, Zhou M (2006). Peritumoral lymphatic invasion is associated with regional lymph node metastases in prostate adenocarcinoma. Mod Pathol.

[4] Longatto-Filho A, Pinheiro C, Pereira SM, Etlinger D, Moreira MA, Jube LF, Queiroz GS, Baltazar F, Schmitt FC (2007). Lymphatic vessel density and epithelial D2-40 immunoreactivity in pre-invasive and invasive lesions of the uterine cervix. Gynecol Oncol.

[5] El-Gohary YM, Metwally G, Saad RS, Robinson MJ, Mesko T, Poppiti RJ (2008). Prognostic significance of intratumoral and peritumoral lymphatic density and blood vessel density in invasive breast carcinomas. Am J Clin Pathol.

[6] Maula SM, Luukkaa M, Grenman R, Jackson D, Jalkanen S, Ristamaki R (2003). Intratumoral lymphatics are essential for the metastatic spread and prognosis in squamous cell carcinomas of the head and neck region. Cancer Res.

[7] Cao F, Hu YW, Li P, Liu Y, Wang K, Ma L, Li PF, Ni CR, Ding HZ (2013). Lymphangiogenic and angiogenic microvessel density in chinese patients with gastric carcinoma: correlation with clinicopathologic parameters and prognosis. Asian Pac J Cancer Prev.

[8] Juttner S, Wissmann C, Jons T, Vieth M, Hertel J, Gretschel S, Schlag PM, Kemmner W, Hocker M (2006). Vascular endothelial growth factor-D and its receptor VEGFR-3: two novel independent prognostic markers in gastric adenocarcinoma. J Clin Oncol.

[9] Tammela T, Alitalo K (2010). Lymphangiogenesis: molecular mechanisms and future promise. Cell.

[10] Wu Y, Grabsch H, Ivanova T, Tan IB, Murray J, Ooi CH, Wright AI, West NP, Hutchins GG, Wu J (2013). Comprehensive genomic meta-analysis identifies intra-tumoural stroma as a predictor of survival in patients with gastric cancer. Gut.

[11] Caner Genome Atlas Research Network, et al. Comprehensive molecular characterization of gastric adenocarcinoma. Nature. 2014;513(7517):202–9. 10.1038/nature13480 PMC4170219 25079317

[12] Massague J (2008). TGFbeta in Cancer. Cell.

[13] Ikushima H, Miyazono K (2010). TGFbeta signalling: a complex web in cancer progression. Nat Rev Cancer.

[14] Ding L, Chen X, Jing K, Wang H, Zhang W (2006). Inhibition of the VEGF expression and cell growth in hepatocellular carcinoma by blocking HIF-1alpha and Smad3 binding site in VEGF promoter. J Huazhong Univ Sci Technolog Med Sci.

[15] Liu D, Li L, Zhang XX, Wan DY, Xi BX, Hu Z, Ding WC, Zhu D, Wang XL, Wang W (2014). SIX1 promotes tumor lymphangiogenesis by coordinating TGFbeta signals that increase expression of VEGF-C. Cancer Res.

[16] Seystahl K, Tritschler I, Szabo E, Tabatabai G, Weller M (2015). Differential regulation of TGF-beta-induced, ALK-5-mediated VEGF release by SMAD2/3 versus SMAD1/5/8 signaling in glioblastoma. Neuro-oncology.

[17] Park KC, Kim SW, Jeon JY, Jo AR, Choi HJ, Kim J, Lee HG, Kim Y, Mills GB, Noh SH (2018). Survival of Cancer stem-like cells under metabolic stress via CaMK2alpha-mediated upregulation of Sarco/endoplasmic reticulum calcium ATPase expression. Clin Cancer Res.

[18] Cheong JH, Park ES, Liang J, Dennison JB, Tsavachidou D, Nguyen-Charles C, Wa Cheng K, Hall H, Zhang D, Lu Y (2011). Dual inhibition of tumor energy pathway by 2-deoxyglucose and metformin is effective against a broad spectrum of preclinical cancer models. Mol Cancer Ther.

[19] Calon A, Lonardo E, Berenguer-Llergo A, Espinet E, Hernando-Momblona X, Iglesias M, Sevillano M, Palomo-Ponce S, Tauriello DV, Byrom D (2015). Stromal gene expression defines poor-prognosis subtypes in colorectal cancer. Nat Genet.

[20] Giannelli G, Villa E, Lahn M (2014). Transforming growth factor-beta as a therapeutic target in hepatocellular carcinoma. Cancer Res.

[21] Holmgaard RB, Schaer DA, Li Y, Castaneda SP, Murphy MY, Xu X, Inigo I, Dobkin J, Manro JR, Iversen PW (2018). Targeting the TGFbeta pathway with galunisertib, a TGFbetaRI small molecule inhibitor, promotes anti-tumor immunity leading to durable, complete responses, as monotherapy and in combination with checkpoint blockade. J Immunother Cancer.

[22] Achyut BR, Yang L (2011). Transforming growth factor-beta in the gastrointestinal and hepatic tumor microenvironment. Gastroenterology.

[23] Suzuki Y, Ito Y, Mizuno M, Kinashi H, Sawai A, Noda Y, Mizuno T, Shimizu H, Fujita Y, Matsui K (2012). Transforming growth factor-beta induces vascular endothelial growth factor-C expression leading to lymphangiogenesis in rat unilateral ureteral obstruction. Kidney Int.

