# Supplementary figures and images for: VEGF-C induced by TGF- β1 signaling in gastric cancer enhances tumor-induced lymphangiogenesis
Source: BMC Cancer. 2019 Aug 13;19:799. doi: 10.1186/s12885-019-5972-y (PMC6692962; doi:10.1186/s12885-019-5972-y)

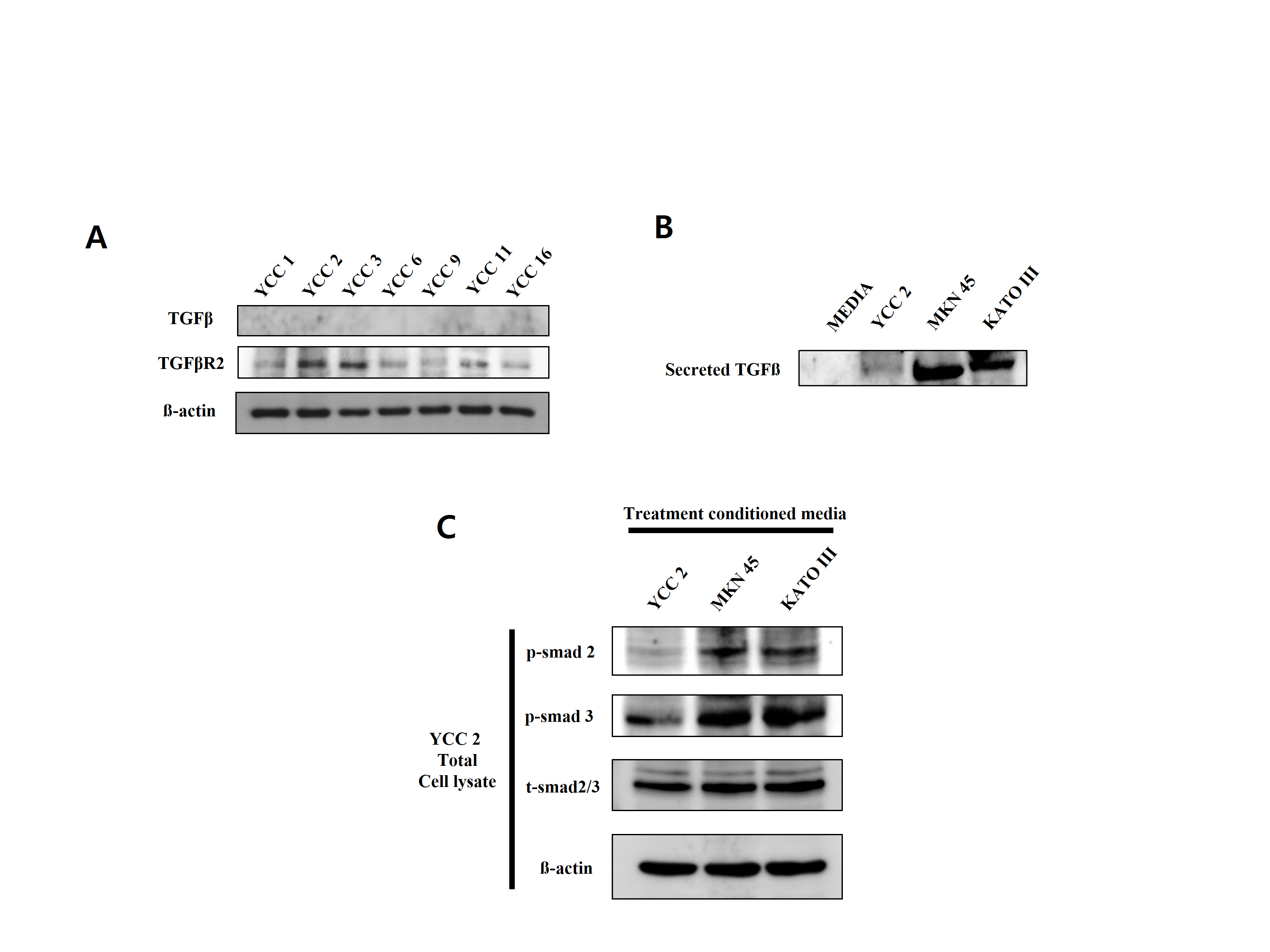

Supplement: Supplementary file 1 — Figure S1 Paracrine regulation of TGF-β1. (A-B) YCC2 was selected as a paracrine model of TGF-β1 production. (C) Smads were detected only in the total cell lysate of YCC2, which was cultured in the conditioned media of MKN45 and KATOIII cells. (TIF 945 kb) [file 12885_2019_5972_MOESM1_ESM.tif]

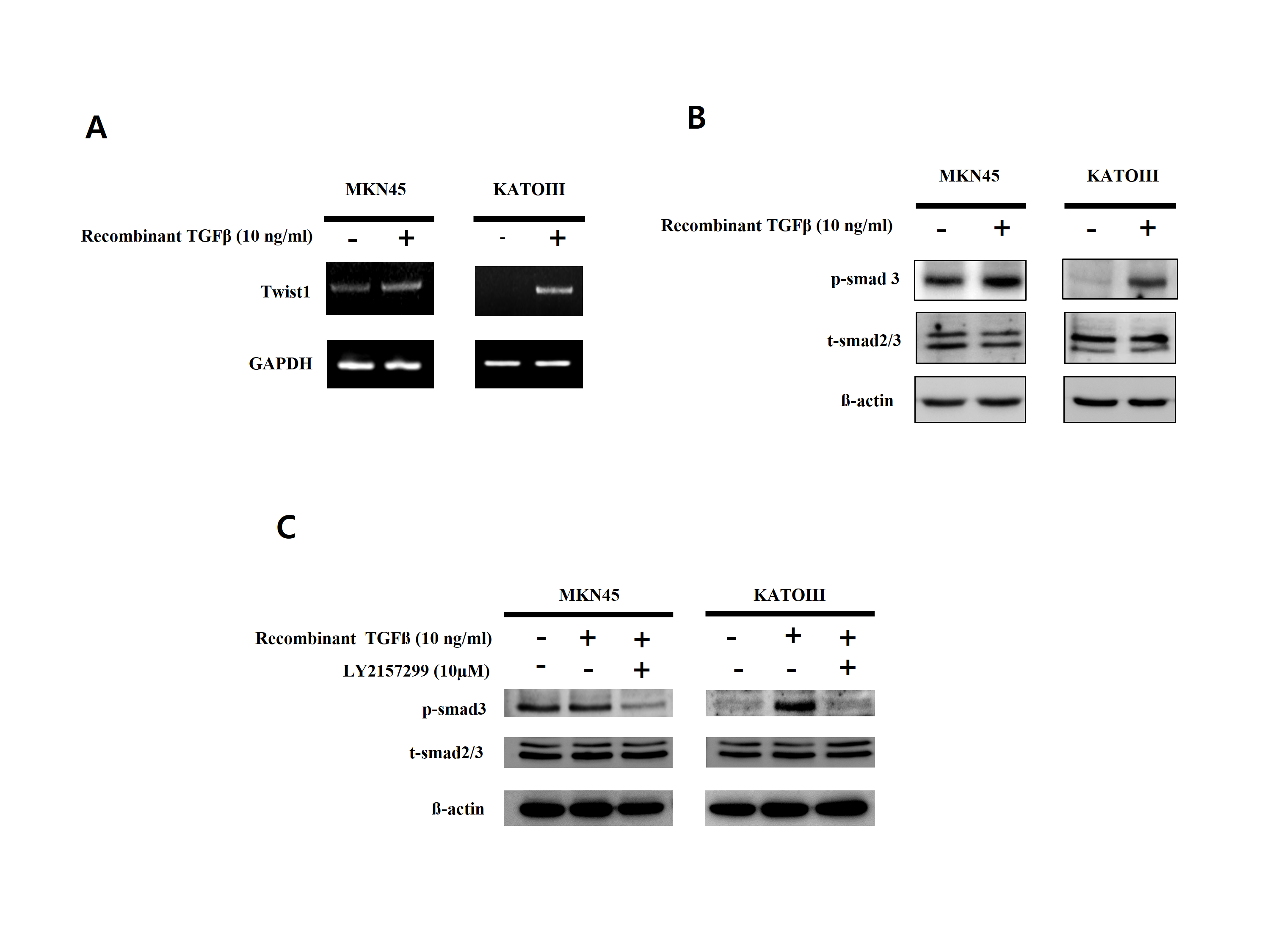

Supplement: Supplementary file 2 — Figure S2 Activated TGF-β1 signaling in MKN45 and KATOIII gastric cancer cells The expression of twist I (A) and p-Smad3 (B) were enhanced in responding to TGF-β1, but was decreased at TGF-β1 receptor inhibitor (LY2157299) (C). (TIF 931 kb) [file 12885_2019_5972_MOESM2_ESM.tif]

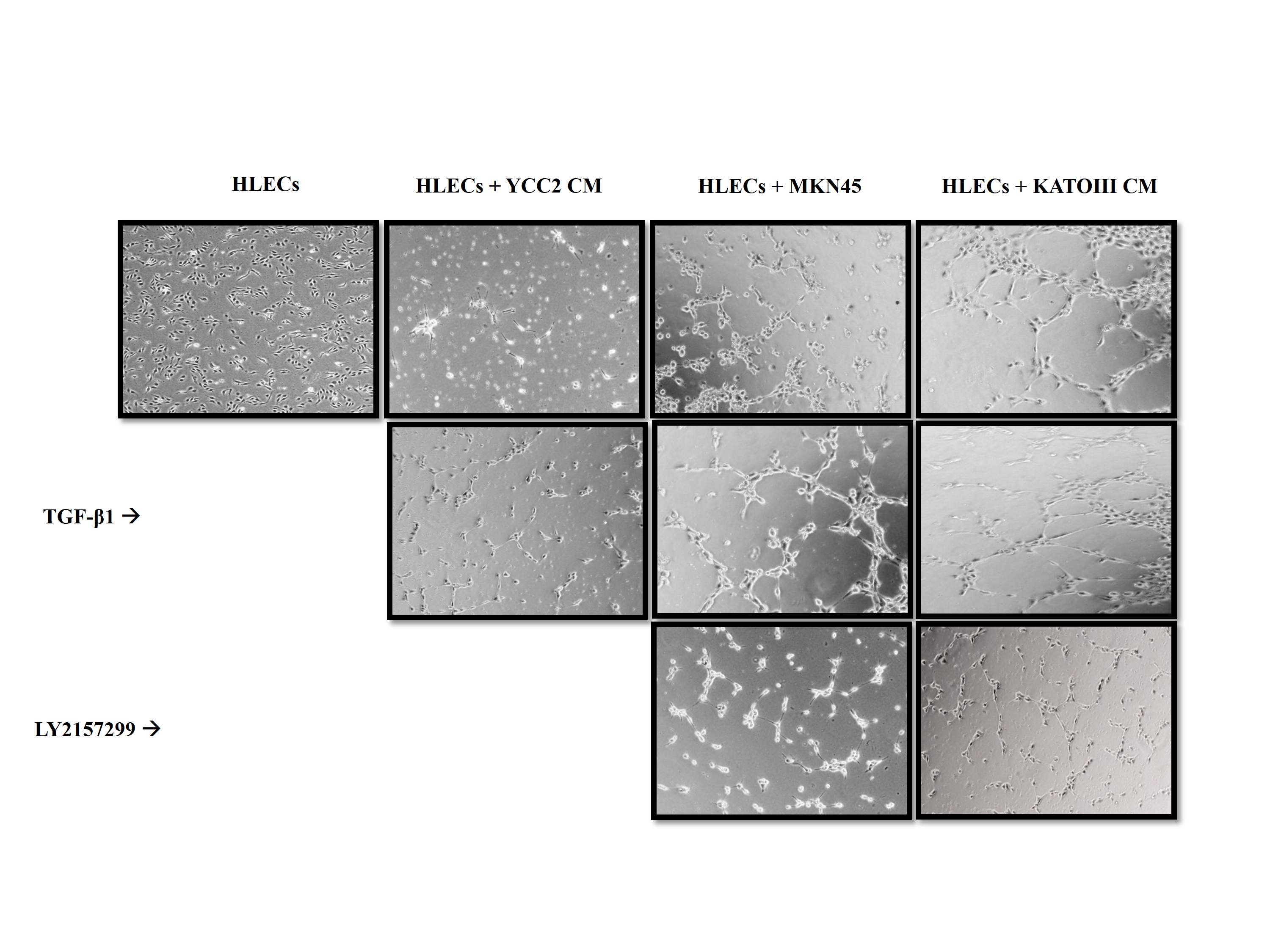

Supplement: Supplementary file 3 — Figure S3 Lymphatic endothelial cell (HLEC) growth in the conditioned media of gastric cancer cells. The growth of HLEC in MKN45- and KATOIII-conditioned media was increased compared to HLECs alone or with YCC2-conditioned media. However, tube formation was decreased in responding to TGF receptor I inhibitor. All photos were taken after 8 h of culture. CM, conditioned media; TβR1 inh., TGF-β receptor 1 inhibitor. (TIF 3336 kb) [file 12885_2019_5972_MOESM3_ESM.tif]

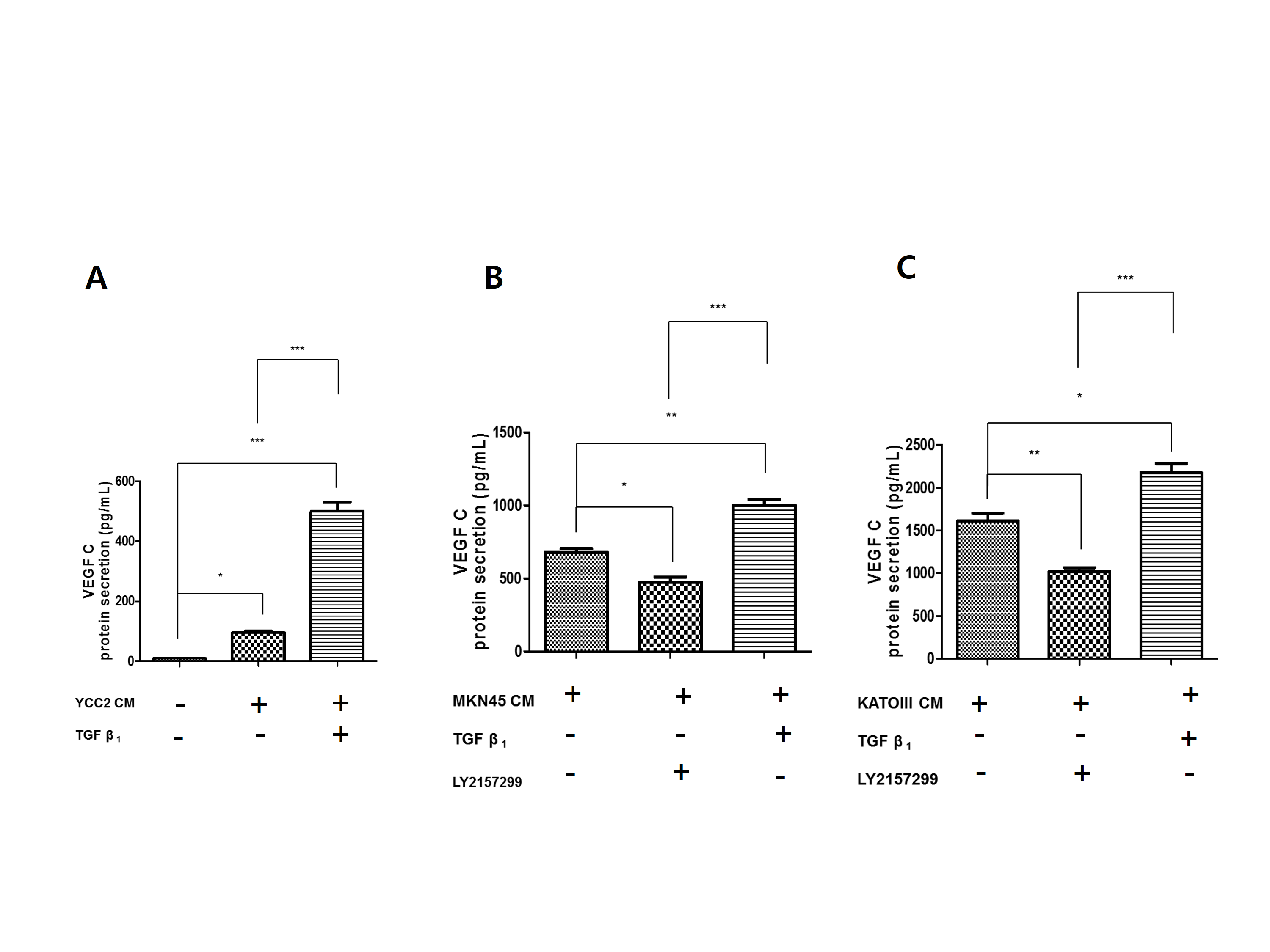

Supplement: Supplementary file 4 — Figure S4 The level of VEGF-C in the conditioned media of gastric cancer cell lines. (A) YCC 2-conditioned media resulted in an increased level of VEGF-C with TGF-β1. (B-D) The level of VEGF-C was increased with TGF-β1, but decreased with TβR1 inhibitor in MKN45- and KATOIII-conditioned media treatments. TβR1, TGF-β receptor 1. *P < 0.05, **P < 0.01, ***P < 0.001, One-ANOVA test). (TIF 871 kb) [file 12885_2019_5972_MOESM4_ESM.tif]
